# Evaluating spatial access to primary care and health disparities in a rural district of Sri Lanka: Implications for strategic health policy interventions

**DOI:** 10.1371/journal.pgph.0005192

**Published:** 2025-09-11

**Authors:** Parami Abeyrathna, Manjula Weerasinghe, Suneth Buddhika Agampodi, Shyamalee Samaranayake, Pahala Hangidi Gedara Janaka Pushpakumara

**Affiliations:** 1 Department of Family Medicine, Faculty of Medicine and Allied Sciences, Rajarata University of Sri Lanka, Anuradhapura, Sri Lanka; 2 Department of Community Medicine, Faculty of Medicine and Allied Sciences, Rajarata University of Sri Lanka, Anuradhapura, Sri Lanka; 3 Center for Public Health, Anuradhapura, Sri Lanka; 4 Department of Family Medicine, Faculty of Medical Sciences, University of Sri Jayawardhanapura, Nugegoda, Sri Lanka; University of Cape Town, SOUTH AFRICA

## Abstract

Primary care accessibility is optimised by equity in service coverage, especially in resource-limited settings. This study examined spatial accessibility to private and public primary care facilities (PCFs) in the Anuradhapura District, Sri Lanka, which offer both allopathic and alternative medicine, while analysing the correlation to social development indices. A two-step floating catchment area (2SFCA) was applied to evaluate spatial accessibility across 657 Grama Niladhari Divisions (GNDs). Data on population, primary care doctors (PCDs), social development indices, and spatial administrative maps were collected from corresponding departments. The Spatial Accessibility Index (SAI) was analysed among 404 PCFs in the ArcGIS application and expressed as the number of PCDs per 10,000 population within a designated buffer (5km/ 10km). SAIs were correlated with the district’s key social development indices. The study found that the private allopathic sector covered 63.7% of PCFs and 49% of PCDs. The national primary care coverage (NPCC) target of one PCD:5000 population was met at 86% by including all allopathic PCDs, but reduced to 25% with only the public sector. The average SAI for a GND was 4.50 and 4.67 for both buffers, indicating sufficient primary care accessibility compared to NPCC targets. SAIs were positively correlated with population density (r[21]=.735, p < 0.01), availability of education facilities (r[21]=.600, p < 0.01), inward healthcare capacity (r[21]=.810, p < 0.001), and availability financial infrastructure (r[21]=.572, p = 0.05). A negative correlation was reported for poverty measures (r[21]=-.603, p = 0.03). The study identified adequate access to primary care in the district, highlighting the private sector’s vital role in service delivery. However, only one-quarter of the NPCC target is provided by public-sector allopathic PCDs. Areas with high population density and educational resources show better access, while poverty is linked to reduced access. A comprehensive approach that addresses both spatial and aspatial factors is necessary to enhance rural healthcare access.

## Introduction

Universal health coverage, the ability of every individual in the community to access high-quality health services without financial hardship, is a goal that can be met through an efficient and equitable primary healthcare system [1]. The primary care domain, serving as the fundamental unit for delivering integrated healthcare services, offers first-contact accessibility to the health system [2]. Good access to primary healthcare is important in achieving universal health coverage, and it is not just a possibility but a proven reality. Countries with strong primary healthcare (PHC) systems experience lower hospital admission rates and better chronic disease management [3,4]. Good primary care is associated with lower mortality rates due to the early detection and prevention of illnesses by early interventions, making it a critical component in pursuing universal health coverage [5],

The World Bank predicts that five billion people will still be unable to access health care in 2030 due to current observations of healthcare inequalities [6,7]. Even high and upper-middle-income countries have demonstrated limited primary care access despite their strategies for developing infrastructure due to workforce shortages and geographic disparities [8]. The fragmentation between public and private healthcare systems observed in developed countries has led to inefficiencies, making primary care access inconsistent [9]. Most low-income countries similarly report limited access after years of investments in expanding health systems and public health initiatives [10,11] due to economic constraints, limited infrastructure, geographic barriers, transportation issues, and cultural beliefs [12–15]. This global challenge underscores the urgency and importance of understanding and analysing the current distribution of healthcare resources and services, and the need to identify and resolve disparities.

Sri Lanka is considered a low-middle-income country with good maternal and child healthcare indicators over the past years due to the prevailing robust PHC system [16]. Sri Lanka reported a high institutional delivery rate of around 99.9%, ensuring safer deliveries and reducing maternal mortality [17]. A publicly-funded universal healthcare policy drives Sri Lanka’s health system without restriction on access [18]. It has a PHC in which a mixture of medical practitioners from allopathic, Ayurveda, Yunani, Siddha, Homeopathic and indigenous medicine or ‘Deshiya Chithsa’ are involved in healthcare delivery [19]. Traditional medicine is often confused with Ayurveda practice, but the latter is identified as a separate entity as recognised by the Ayurveda Council in Sri Lanka. Ayurveda is the most dominant traditional medical system in Sri Lanka [20]. ‘Deshiya Chikithsa’ is based mainly on a family-inherited knowledge system that is gained through experience. Sidda and Unani are mostly practised among ethnic minorities. Due to the pluralistic and multi-sectorial nature of the health system in Sri Lanka, many healthcare seekers reported regional and ethnic health disparities [21]. Rural areas experience limited access to specialised healthcare services, leading to disparities in treatment availability [21]. While Sri Lanka has a strong PHC system, reliance on private healthcare has increased, particularly among urban populations, exacerbating financial barriers for lower-income groups [22].

Preventive PHC in Sri Lanka focuses on disease prevention, health promotion, and early intervention to improve overall public health. It is delivered through a network of Medical Officers of Health (MOH) areas, ensuring accessibility at the community level. Primary care services are mainly focused on the diagnosis, treatment, and management of diseases at the community level through three levels. The primary level of care provides outpatient and inpatient care for minor ailments and early-stage diseases through Divisional Hospitals (DH) and Primary Medical Care Units (PMCUs, previously known as Central Dispensaries). At the secondary level of care, they provide outpatient specialised medical services through District General Hospitals and Base Hospitals (BH). The tertiary level of care consists of outpatient services at advanced hospitals with specialised consultative services, such as in Teaching Hospitals (TH), Provincial Hospitals (PH) and National Hospitals. The private sector PCFs comprise private hospitals with OPD services and private allopathic practitioners, often called General Practitioners (GPs), who are mostly the public medical officers practising in the community outside their regular working hours [23].

The Ministry of Health (MoH) of Sri Lanka proposed new health reforms in 2017 to resolve existing discrepancies in PHC and build the capacity of primary care delivery to achieve expected universal health coverage targets [24]. In 2018, the “Primary Healthcare-System-Strengthening Project (PHSSP)’ was initiated [25]. The project focused on three thematic areas: reorganisation of the PHC by defining the catchment area and population for each PMCU; strengthening the PMCUs with trained staffing, optimising drug and expanding laboratory services; and establishing a technology-based health management information system to provide electronic personal health records. This project introduced ‘shared care cluster system’ which allocated one allopathic PCF to a population cluster, including several GNDs. Several of these PCFs were linkd to an apex hospital to formulate a “cluster”. PHSSP mainly concentrated on preventive aspects with screening of assigned population for NCDs and promoting utilization of healthy lifestyle clinics. However, with the COVID-19 outbreak and post-COVID financial crisis, the project has slowed down. With the funding of the world bank, a new project was initiated in 2025, named “Primary Healthcare System Enhancing Project” (PHSEP), focusing mainly on curative primary care.

Primary care access has not been previously evaluated in Sri Lanka except for healthcare utilisation surveys at the national level [26]. A spatial approach to assessing healthcare accessibility is crucial because it provides a geographically informed perspective on disparities in healthcare access [27].

Anuradhapura, the largest district in Sri Lanka, but has a relatively low population density of 120 people per square kilometre compared to an urban district like Colombo, with 3,666 people per square kilometre [28]. The district has 22 Divisional Secretariat Divisions (DSDs), with a 96% rural population out of 953,162. [29]. These DSDs are further divided into 694 Grama Niladhari Divisions (GNDs), the smallest administrative units. The Anuradhapura Urban Council governs 25 GNDs, while rural GNDs fall under Divisional Secretariats (Pradeshiya Sabha). The economy is primarily agricultural, with most residents involved in crop production and livestock farming. Public transport, mainly buses, is the key means of accessing healthcare. While the central town areas have a well-developed road network, rural roads are less maintained. [30]. Anuradhapura district has the highest chronic kidney disease burden in Sri Lanka, leading to significant out-of-pocket healthcare expenditures for patients, exacerbating their economic challenges.[31,32]. This study examined the spatial distribution and accessibility of primary care services in the Anuradhapura district of Sri Lanka, while actively highlighting disparities in primary care in relation to key social development indices.

## Methods

### Study design and setting

This spatial analytical study was conducted between April 2021 and December 2021 in the Anuradhapura district of Sri Lanka. As in the rest of Sri Lanka, most of the population in Anuradhapura relies on the allopathic healthcare system for their medical needs [19]. Both the public and private sectors provide primary care services. Primary care provided by complementary and alternative medicinal (CAM) practices is served mainly through a network of public-funded Ayurveda hospitals [20]. Private PCFs of CAM included indigenous medical practices and several other practices of Ayurveda, Yunani, Siddha, and Homeopathy practitioners scattered in the rural remotes and suburban regions of Anuradhapura district [33]. Anuradhapura urban council area comprises the tertiary-level Teaching Hospital with a large-scale OPD. The need for an exensive collection of current practices of private sector PCFs was highlighted due to unavailability of a proper registry or database for reference.

### Ethics statement

This sub-study, as part of the more extensive research titled ‘The Exploration of Primary Care Accessibility, Utilisation Patterns, and Morbidity Profiles of Primary Health Care Seekers in Anuradhapura District of Sri Lanka,’ received ethical approval from the Ethics Review Committee of the Faculty of Medicine & Allied Sciences, Rajarata University of Sri Lanka (ERC/2020/66) for the entire project, including all sub-components.. The, informed written consent was obtained for other components of the study. Secondary statistical data from the departments such as Survey, Provincial Director of Health and Regional Director of Health were obtained with administrative clearance from the respective heads of the institutes. The GPS location data of PCFs were collected from publicly available and accessible noticeboards indicating the presence of allopathic or CAM PCFs in the district. Except for the GPS location and type, no other person-identifiable data was collected from any doctor or person in the community.

### Data sources and collection

We collected spatial data of the PCFs and district maps through the Global Positioning System (GPS) and Department of Survey, respectively. Spatial data like the number of PCDS and DSD-level socio-demographic data were collected from official databases in different departments, such as the Provincial Department of Health, Provincial Ayurveda Department, and Census and Statistics (S1 Table).

Sri Lankan health authorities currently lack a publically accessible database on the locations of public and private sector PCFs. We planned the study to collect the exact spatial locations of public and private sector PCFs distributed in the district. The data collectors recorded the spatial locations of PCFs after visiting each facility. We collected the data of public PCFs based on a list issued by the provincial health authorities. In the absence of any record of the private sector PCFs including GPs except for a few registered at the regional director of health services, their GPS locations were explored in an extensive road-based survey across every road in the district. The locations of GPs or CAM practices were recorded if the public display boards at their practices included a registration number from the Sri Lanka Medical Council or Ayurveda Medical Council of Sri Lanka, respectively. The active practices were confirmed by neighbourhood informants like *Gramaseva Niladhari* (the village’s key administrative officer) and villagers. GPS data were coded by a handheld GPS Juno SB Trimble device [34]. The number of available PCDs at the practice and type of practice was updated in an Excel sheet. No information on individually identifiable PCDs was included.

We collected the most recently updated population data of GNDs and DSDs from the Department of Census and Statistics [28]. The key social development indices at the DSD level were referred to from the District Statistical Handbook 2021 on the Department of Census and Statistics website [29]. The Department of Survey, Sri Lanka, provided spatial administrative maps of GNDs and DSDs of the district with population data.

### Variable selection

The Spatial Accessibility Index (SAI) was calculated as a measure of accessibility based on variables such as the type of primary care provider (allopathic or CAM) and the service provision sector (public or private).

We explored a bivariate correlation between the SAI and key social developmental indices available at the DSD level [35]. Social developmental indices are quantitative and qualitative changes in the economy that measure the standard of living and economic health of a specific area, including social, environmental, and any other kinds of development. The key social developmental factors, categorised as population and vital statistics, healthcare infrastructure, educational infrastructure, and financial infrastructure, in the analysis are referred in S2 Table.

The district’s urban population was identified based on the administrative definition of Sri Lanka, which recognises areas under the urban council as ‘urban’ [36]. Anuradhapura town area is the only urban council in the Anuradhapura district. Other DSDs are regulated by local administrative councils called ‘*Pradeshiya Sabha’.* Each DSD has a unique number of GNDs ranging from 15 to 43.

### Data analysis

The data analysis was conducted in two phases:

1. Geographic Information System (GIS)-based spatial accessibility analysis
2. Descriptive and inferential statistics

#### 1. GIS-based spatial accessibility analysis.

**Fig 1:**
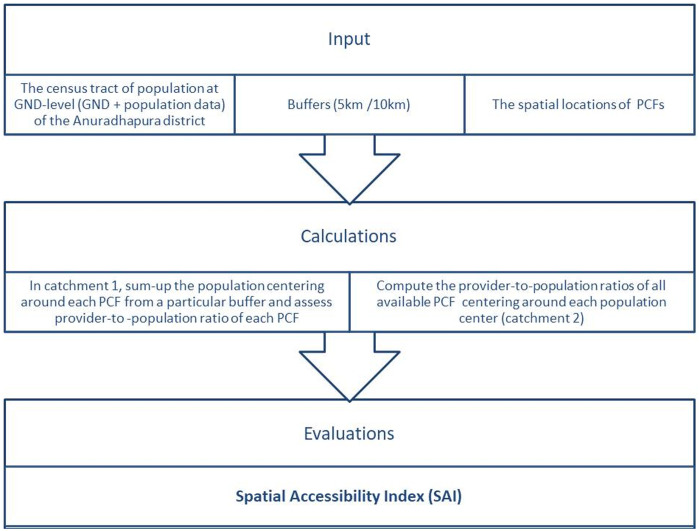
Shows the basic 2SFCA methodology and the evaluations in the study.

Primary care coverage in the district was assessed using the Two-Steps-Floating-Catchment-Area (2SFCA) method in the ArcGIS version 10.5 software [37]. The 2SFCA method is a widely used spatial accessibility measure, particularly in healthcare research. It analyses the spatial distribution of both the supply side (healthcare facilities) and the demand side (population) to evaluate accessibility, as summarised in Fig 1.

Recent advancements in the 2SFCA method include distance decay effects, variable catchment sizes, hierarchical 2SFCA (H2SFCA), and comparative analysis models. These techniques require advanced technical expertise and additional data resources, which are challenging in low-income study settings.

The spatial accessibility index (SAI) is typically calculated as the ratio of healthcare providers to the population within a defined geographic area, incorporating factors such as distance and availability of services [27].

This study used Euclidean/ spatial distances to estimate SAI instead of travel distance due to technical limitations in adapting the available road network data into ArcGIS and the unavailability of spatial road network maps at many rural-remote GNDs in free sources.

Essentially, 2SFCA has two steps: each creates an area of coverage called a catchment in ArcGIS over the basemap layer of the Anuradhapura District GNDs and administrative boundaries.

Spatial Accessibility Index (SAI) Calculation:

Step 1: A buffer zone (Catchment 1) was created around each PCF, summing the population reachable within the area. The provider-to-population ratio was computed based on the field capacity of PCDs in each PCF.

Step 2: Another buffer zone (Catchment 2) was established around population centres (GNDs). The provider-to-population ratios from PCFs within this buffer were summed to calculate the SAI, indicating healthcare accessibility.

The first step focuses on individual healthcare facilities, offering a facility-centered assessment of accessibility. The second step shifts focus to population access, refining the results by aggregating the influence of multiple facilities.

The two catchments lay on top of one another, or ‘floating’, and adjust according to the availability of services and population density. Census tract centroids were created to mark the population centres of each GND.

According to the Ministry of Health, Sri Lanka (MoH) reports in 2019, a healthcare facility was theoretically available at every 5km travel distance in the country [38]. Thus, 5km and 10km buffers were used in the 2SFCA method to compare the spatial coverages between public, private, allopathic, and CAM practices. SAI at the DSDs were estimated by measuring the GNDs’ mean in each DSD. For the simplified interpretability, SAI is expressed as the number of PCDs per 10,000 people within a particular catchment.

#### 2. Descriptive and inferential statistics.

The PCDs were categorised by sector, indicating whether they were part of the public or private sector, and by medical practice type, whether allopathic or complementary and alternative medicine (CAM). GNDs were classified based on geographical location, distinguishing between urban and rural areas. Descriptive statistics was used to describe the composition of the PCDs and PCFs in each subcategory. The mean SAI at the DSD level and the social development indices (2021) were used to perform inferential statistics (independent sample t-test and bivariate correlations) using the Statistical Package for Social Sciences (IBM SPSS), Version 25.0.

## Results

### 1. Spatial distribution of primary care services

In the public allopathic sector, divisional hospital Type C predominantly delivered primary care services, which housed 22 PCFs (5.5% of the total) and 44 PCDs (Table 1).

**Table 1:** An Overview of primary care facilities and primary care doctors in Anuradhapura District, Sri Lanka.

	Primary care facility	No of facilities (%)	No of primary care doctors (%)
**Public allopathic sector**			
	**Teaching Hospital of Anuradhapura**	1 (0.3)	20 (4)
	**Base hospital**	6 (1.5)	30 (6)
	**Divisional hospital-A**	2 (0.5)	8 (2)
	**Divisional hospital-B**	9 (2.2)	27 (5)
	**Divisional hospital-C**	22 (5.5)	44 (8)
	**PMCU**	21 (5.2)	21 (4)
	**Total**	61 (15.2)	150 (29)
**Private allopathic sector**			
	**Private allopathic/GPs**	257 (63.7)	257 (49)
	**Total**	257 (63.7)	257 (49)
**Public Ayurveda sector**			
	**Public Ayurveda hospitals with OPDs and MOH centres**	25 (6.2)	56 (11)
	**Total**	25 (6.2)	56 (11)
**Private sector: Complementary and Alternative Medicine (CAM)**			
	**Private CAM practitioners**	60 (14.8)	60 (11)
	**Total**	60 (14.8)	60 (11)
**Overall total**		**403(100)**	**523(100)**

OPD: Outpatient Departments; PMCU: Primary Medical Care Units; MOH: Medical Officer of Health; GP: General Practitioners.

In total, the district had 403 PCFs and 523 PCDs. Private allopathic providers played a central role, constituting 63.7% of PCFs and 49% of PCDs. The public Ayurveda sector contributed with 25 PCFs (6.2%) and 56 PCDs (11%), while the private CAM practices included 60 PCFs (14.8%) and 60 PCDs (11%).

**Fig 2:**
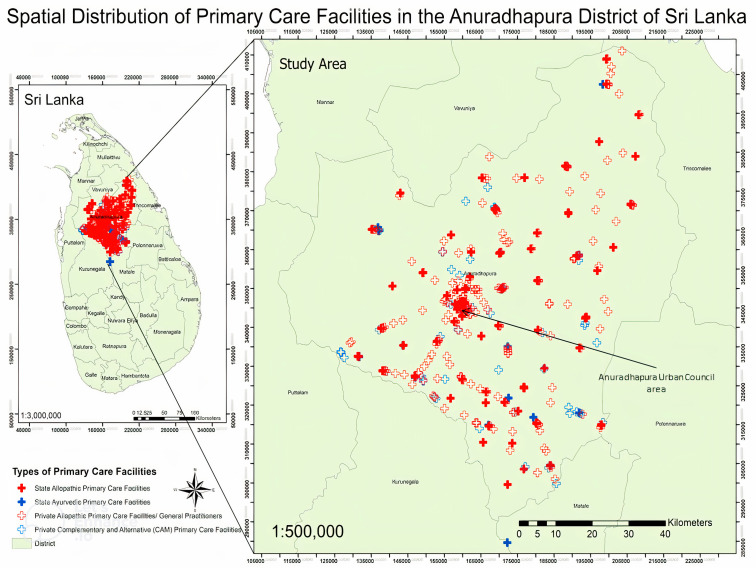
Spatial distributions of primary care facilities in Anuradhapura district of Sri Lanka.

As shown in Fig 2, most PCFs were located close to urbanised regions and the suburbs in the Anuradhapura district.

The map depicts Primary Care Facilities (PCFs) in the Anuradhapura District of Sri Lanka, categorised by type and ownership. District boundaries are outlined in green.

The basemaps used in this study were sourced from the Department of Survey, Sri Lanka (https://survey.gov.lk/sdweb/home.php) for research purposes only. All rights remain with the Department of Survey, and any errors in interpretation or analysis are solely those of the authors.

### Spatial Accessibility Index (SAI)

**Fig 3:**
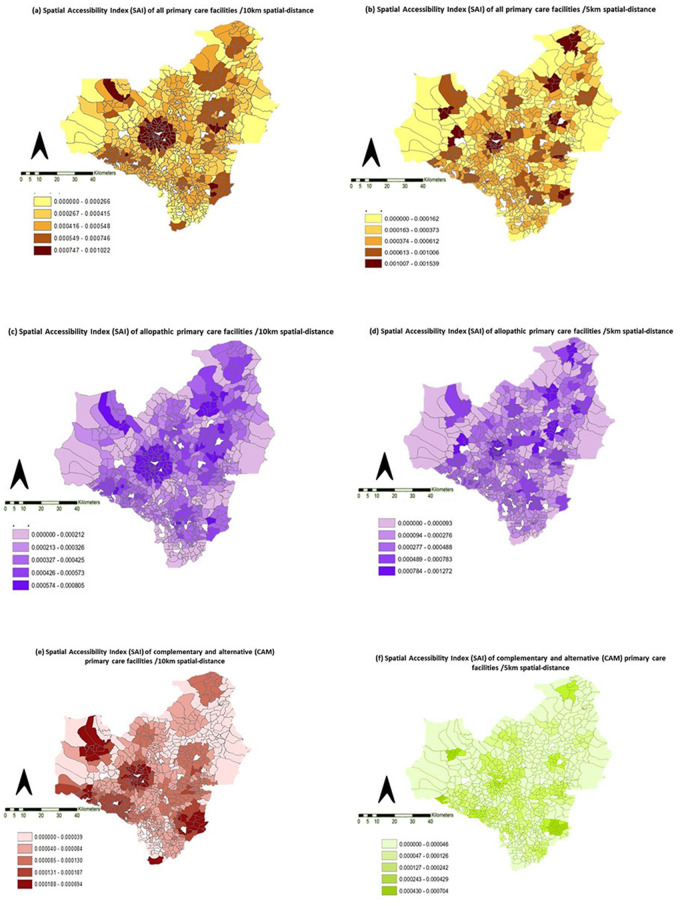
Spatial distribution of Spatial Accessibility Index (SAI) of different types of primary care facilities in Anuradhapura district of Sri Lanka. The maps illustrate the Spatial Accessibility Index (SAI) for 5km and 10km buffers for primary care facilities. These buffers define accessibility, representing common travel methods like walking or public transport. (a) SAI for all primary care facilities (10km): Light Yellow to Dark Brown, showing increasing accessibility. Range: 0.000000 to 0.001022., (b) SAI for all primary care facilities (5km): Light Yellow to Dark Brown, increasing accessibility. Range: 0.000000 to 0.001539, (c) SAI for allopathic facilities (10km): Light Purple to Dark Purple, increasing accessibility. Range: 0.000000 to 0.000805, (d) SAI for allopathic facilities (5km): Light Purple to Dark Purple, increasing accessibility. Range: 0.000000 to 0.001272, (e) SAI for CAM facilities (10km): Light Red to Dark Red, increasing accessibility. Range: 0.000000 to 0.000694, (f) SAI for CAM facilities (5km): Light Green to Dark Green, increasing accessibility. Range: 0.000000 to 0.000704.

The spatial maps of different types of PCFs and catchments further showed the district’s inequitable distribution of primary care (Fig 3).

The basemaps used in this study were sourced from the Department of Survey, Sri Lanka (https://survey.gov.lk/sdweb/home.php) for research purposes only. All rights remain with the Department of Survey, and any errors in interpretation or analysis are solely those of the authors.

PCFs located close to the district border were scarce and scattered throughout. Lower SAIs were more towards rural areas and district borders. One-fourth of the population lived in areas with the lowest primary care accessibility (SAI < 1.72/10,000). An Independent Samples t-test revealed a significant difference in the mean SAI between GNDs in urban areas (n = 25, M = 8.8/10,000, 2SD = 1/10,000) and rural remote areas (n = 632, M = 4.5/10,000, 2SD = 3.8/10,000), t(655)=11, p < 0.001.

The national target for primary care coverage (NPCC) of one PCD to 5000 population (if both allopathic and CAM are included) ratio was achieved in 70% (n = 464) and 92% (n = 605) of GNDs within 5km and 10km buffers respectively. The coverage of one allopathic PCD to 5000 population ratio was achieved in 64% (n = 420) and 86% (n = 565) GNDs within 5km and 10km buffers. Individual public and private allopathic PCDs contributed 25% (n = 166) and 56% (n = 368) of GNDs to achieve NPCC in a 10km buffer (S1 Fig).

### SAI and the relationship with key social development indices

This study assessed the bivariate correlation between the key social development indices and the mean SAI at the DSD level (S3 Table). Positive correlations of SAI were observed for population density (r[21]=.735, p < 0.01) and the availability of education facilities such as schools with bilingual curriculum (r[21]=.600, p < 0.01); availability of healthcare facilities with inward beds (r[21]=.810, p < 0.001) and annual OPD admissions (r[21]=.643, p = 0.01); availability of financial infrastructures such as restaurants and canteens (r[21]=.572, p = 0.05 and housing quality indicators such as households with Asbestos roofs (r[21]=.602, p = 0.03). Strongly negative correlations were reported for poverty measures: proportion of households receiving ‘Samurdhi’ aid, a monthly financial aid given to the poorest people in the country (r [21]=-.603, p = 0.03) and employment in agriculture as indicated in the land area of paddy harvest in the district (r [21] = -.530, p = 0.011).

## Discussion

This study is the first to analyse spatial access to primary care services in Sri Lanka, highlighting the private sector’s role as the main provider in both urban and rural areas. It was found that PCFs are concentrated in urban regions, while rural areas face service coverage gaps. The study emphasises the need to evaluate accessibility in relation to social development indices to identify healthcare disparities. The spatial analysis method provides a valuable framework for assessing primary care access across various regions, addressing the country’s technical and resource challenges.

This study highlights the underreported role of the private sector in primary care delivery in rural Sri Lanka [39], emphasizing its impact on healthcare accessibility, especially in urban areas, while raising concerns about affordability and equity for low-income populations [40,41]. Despite the high quality of care in private hospitals, lack of practice registration has led to overlooked contributions of private-sector PCDs in service coverage [42]. The lack of consideration of spatial accessibility has led to a disproportionate division of the healthcare workforce in the health system. The resource constraints, urban-centric development and shortage of human and physical resources would have limited the expansion of public sector PCF in rural Sri Lanka. The widespread presence of private allopathic PCDs reflects the total private allopathic PCFs count rather than their actual working hours in calculating the SAI in the 2SFCA method, as many private GPs work part-time while public sector doctors follow fixed hours (8 am–4 pm). Further research is needed to assess the impact of clinic hours and appointment availability on healthcare spatial access in Sri Lanka.

Our research study reported that only 25% of the district population achieved NPCC via the available public-sector PCDs. NPCC target of one PCD to a population of 5000 was introduced as part of primary healthcare reforms intending to expand primary care services through a proposed “shared care cluster system”, which is now partially implemented in certain districts of the country [24,25]. The rising demand for free of charge public allopathic PCFs lead to overcrowding of OPDs and delivery of insufficient levels in quality of care by PCDs. A significant remodelling of public PCFs is needed to expand healthcare accommodation facilities and human resources to meet the higher demand for free primary care services.

Our study reported that the majority of the GNDs reported a nearby PCF within 5–10 km spatial distances; however, only 14% of the GNDs of the district reported public transport by bus or railway [43]. Despite PCFs’ proximity to GNDs in the district, the efficiency of the public transport networks is inadequate and contributes to indirect medical costs. The out-of-pocket expenditure on transport in the region has been reported to be between 11-14.6% [44,45]. The population spent more costs on travelling to public healthcare facilities than the private sector [46]. The public should develop public transport between service providers in demand and population centres based on population demand. While regulating the direct and indirect medical costs in the private sector, a coordinated collaboration between the public and private sector would be a timely strategy to achieve universal health coverage in low-income settings.

This study observed a significant disparity in primary care access linked to population density, educational facilities and financial infrastructure in urban areas. Urbanised, economically developed areas tend to have better healthcare access as a general concern [47]. The availability of educational facilities influences healthcare access through the availability of educational sources for the community, improving healthcare behaviours and demand [48]. The attraction of doctors depends on the presence of adequate schools for their children, healthcare workforce development in the region, access to banks, financial services, and insurance programs improve both healthcare supply and demand enabling timely medical interventions and attract good healthcare investments [49,50]. Healthcare workforce development is needed to improve spatial healthcare access, and healthcare planning should be integrated during economic development strategies to ensure equitable access.

The urban concentration of PCDs is driven by their presence in secondary and tertiary hospital OPDs and the high-demand private sector offering allopathic and CAM services. In contrast, limited rural primary care access is influenced by community infrastructure and household income, aligning with this study’s findings [51,52]. Healthcare workforce shortages in rural areas are exacerbated by poor retention of PCDs, driven by underdeveloped infrastructure, limited education opportunities, and lack of career growth and earnings [53,54]. The high prevalence of chronic kidney disease in the district, linked to water safety and quality, worsens healthcare disparities by diverting human resources [31]. Healthcare authorities and the labour ministry must tackle this by improving community living standards.

This study highlights healthcare access disparities in rural areas of a lower-middle-income country due to inequitable resource distribution. Research on Malaysia’s dual public-private system shows lower provider-to-population ratios and greater travel burdens in rural areas, leading to healthcare utilization disparities [55]. Similarly, studies in China have demonstrated that transportation infrastructure alone is insufficient to improve healthcare access; instead, a balanced distribution of medical resources is essential [49]. The bi-directional causal relationship between poverty and health has been widely documented [27,56]. Poverty restricts healthcare access through financial barriers, transportation limitations, difficulty securing government appointments, reliance on costly private care, and high travel expenses for specialized treatment. [55]. Higher socio-economic status, including urban residence and higher education, is linked to better healthcare access at both individual and aggregate levels [49,57]. These findings suggest that improving primary care accessibility in the GNDs requires a multifaceted approach, addressing both the expanding primary care facilities in underserved regions through increased government funding and resource allocation [58].

### Limitations

This study was conducted in a rural dominant setting. Thus euclidean distances offer a standardized measure of proximity, enabling regional accessibility comparisons without infrastructure or congestion-related variability across urban and rural territories. Also the study’s baseline assessment helped overcome challenges in technical expertise and data limitations for real time traffic data, road network data and transport schedules in low resource setting.

MOHs were excluded from the study as they had limited roles in primary care, were mainly involved in specific areas in the preventive sector (e.g., maternal and child health), and were not engaged in an open-extended commitment to primary care. The SAI value can be influenced by the edge effect close to district borders [59] and geographical barriers not considered in the analysis [60]. Correlations between SAI and social development indices can be influenced by other confounding factors in the region.

### Conclusion

The significant role of the private allopathic sector in covering PCFs and PCDs is evident, with the majority of PCFs and PCDs being privately operated. The disparity in primary care coverage is notable, with only one-quarter of expected coverage met with public allopathic PCDs. The positive correlations between DS-level SAIs and factors such as population density, education facilities, healthcare capacity, and financial infrastructure underscore the importance of these elements in enhancing primary care accessibility. Conversely, the negative correlation with poverty measures indicates that higher poverty levels are associated with lower accessibility. Future studies needs further evaluations on the impact of travel time, and working hours primary care accessibility and interventions for bridging the gap for primary care coverage through public-private collaboration in the country.

## Supporting information

S1 Table Data sources and method of data collection for primary care facilities and primary care doctors. (PDF)

S2 Table The key social developmental domains, indicators and data availability. (PDF)

S3 Table Correlation matrix showing the relationship between key social developmental indicators and spatial accessibility index within divisional secretariat divisions of Anuradhapura district of Sri Lanka. (XLSX)

S1 Fig Spatial distribution of Grama Niladhari Divisions, which achieved national primary care coverage targets by public and private sector primary care doctors in the Anuradhapura district of Sri Lanka. The basemaps used in this study were sourced from the Department of Survey, Sri Lanka (https://survey.gov.lk/sdweb/home.php) for research purposes only. All rights remain with the Department of Survey, and any errors in interpretation or analysis are solely those of the authors.
(TIF)
