# Supplementary material for: Evaluating spatial access to primary care and health disparities in a rural district of Sri Lanka: Implications for strategic health policy interventions
Source: PLOS Glob Public Health. 2025 Sep 11;5(9):e0005192. doi: 10.1371/journal.pgph.0005192 (PMC12425277; doi:10.1371/journal.pgph.0005192)
Supplement: S1 Table — (PDF) [file pgph.0005192.s001.pdf]

S1 Table

Data Sources and Method of Data Collection for Primary Care Facilities and Primary Care Doctors

Entity	Data sources and collection methods
**State allopathic PCFs and PCDs**	List of PCFs and available PCDs were collected via a physical visit to Provincial Director of Health Services, verified by physical visit for GPS data
**State CAM PCFs and PCDs**	List of PCFs and available PCDs collected via a physical visit to the Provincial Ayurveda Department of North Central Province, verified by physical visit for GPS data
**Private allopathic PCFs and PCDs**	Collection of GPS coordinates by visiting each PCF by a roadway survey and confirmed by the availability of public display boards and key informants of the neighborhood
**Private CAM PCFs and PCDs**	Collection of GPS coordinates by visiting each PCF by a roadway survey and confirmed by the availability of public display boards and key informants of the neighborhood

GPS: Global Positioning System, CAM: Complementary and Alternative Medicine, PCF: Primary Care Facility PCD: Primary Care Doctors
