# Supplementary material for: Evaluating spatial access to primary care and health disparities in a rural district of Sri Lanka: Implications for strategic health policy interventions
Source: PLOS Glob Public Health. 2025 Sep 11;5(9):e0005192. doi: 10.1371/journal.pgph.0005192 (PMC12425277; doi:10.1371/journal.pgph.0005192)
Supplement: S2 Table — (PDF) [file pgph.0005192.s002.pdf]

S2 Table

The Key Social Developmental Domains, Indicators and Data Availability

**Category**	**Variable**	**Operationalization (Definition & Measurement)**	**Measurement Unit**	**Data Source**
Population & Vital Statistics	Population Density	Number of people per square kilometer within each Divisional Secretariat Division (DSD), indicating population concentration and urbanization.	Persons/km^2	Sri Lanka Census
Healthcare Infrastructure	Hospital Beds	Total number of inpatient beds available in hospitals and primary healthcare facilities per district, reflecting treatment capacity.	Beds per 1,000 people	Ministry of Health
	OPD Admissions	Annual outpatient visits recorded in primary care centers, assessing healthcare utilization rates and accessibility.	Admissions/year	Ministry of Health
Educational Infrastructure	Schools with Bilingual Curriculum	Number of schools offering bilingual education (Sinhala & English), linked to improved literacy rates and health awareness.	Schools per DSD	Department of Education
Financial Infrastructure	Number of Banks	Total count of formal banking institutions per region, reflecting financial inclusion and local economic activity.	Institutions per DSD	Central Bank Reports

Category	Variable	Operationalization (Definition & Measurement)	Measurement Unit	Data Source
	Number of Restaurants & Canteens	Number of food establishments serving local and commercial sectors, used as a proxy for urbanization and economic development.	Establishments per DSD	Municipal Reports
Housing & Living Conditions	Households with Asbestos Roofs	Proportion of households with asbestos roofing, indicating socioeconomic status and affordability of quality housing.	Percentage of households	Housing & Urban Development Authority
Poverty Measures	Households Receiving 'Samurdhi' Aid	Percentage of households receiving Samurdhi financial aid, a government support program for low-income groups.	Percentage of households	Ministry of Social Welfare
Employment & Agriculture	Paddy Harvest Land Area	Total land area under paddy cultivation, indicating dependence on agriculture and rural employment levels.	Hectares per district	Agricultural Ministry

GND Grama Niladhari Divisions; DSD Divisional Secretariat Divisions; OPD Outpatient Department; ECCD Early Childhood Care and Development; GCE OL and AL General Certificate of Education (Ordinary Level and Advanced Level); DSD District Secretariat Division
