# Supplementary figures and images for: Evaluating spatial access to primary care and health disparities in a rural district of Sri Lanka: Implications for strategic health policy interventions
Source: PLOS Glob Public Health. 2025 Sep 11;5(9):e0005192. doi: 10.1371/journal.pgph.0005192 (PMC12425277; doi:10.1371/journal.pgph.0005192)

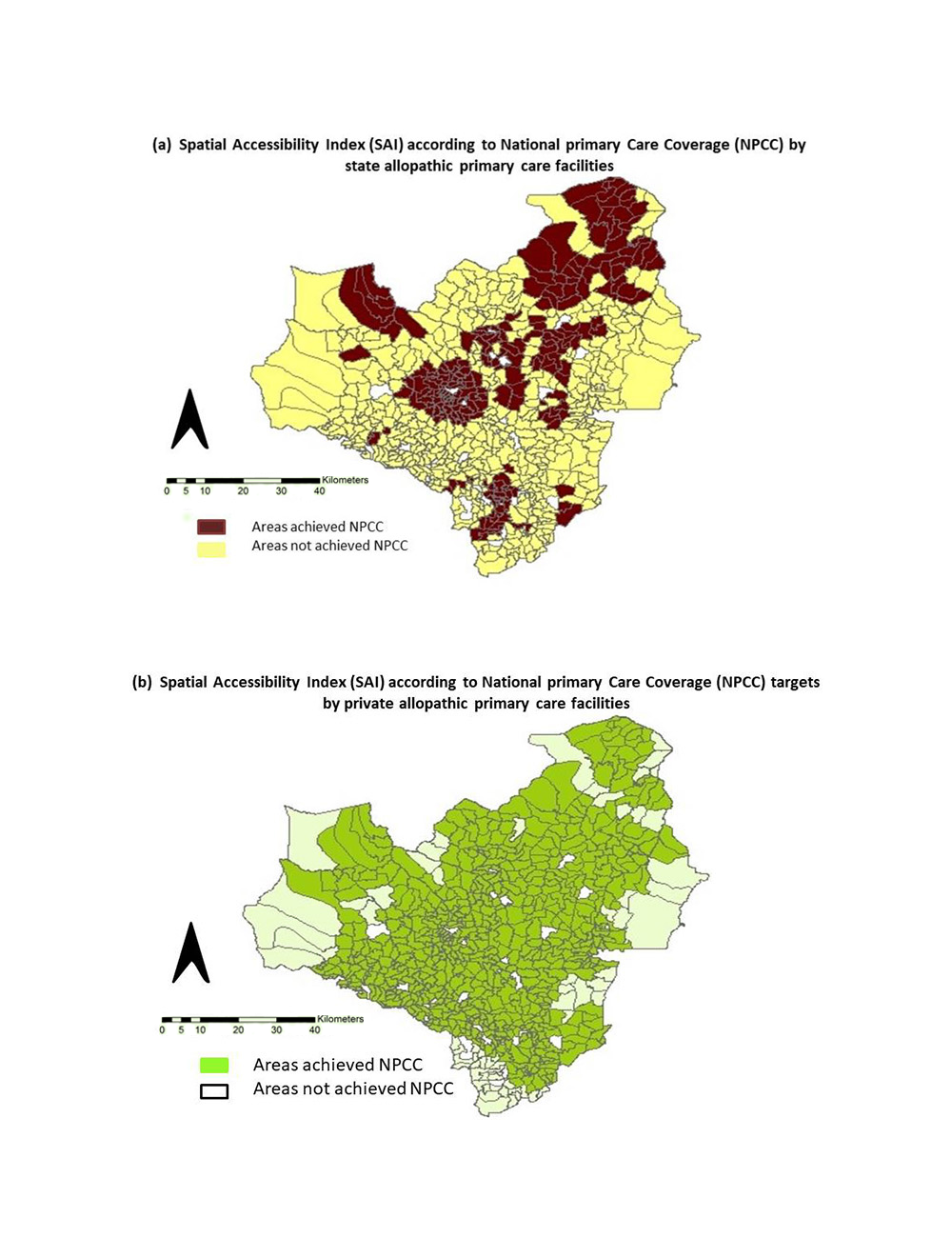

Supplement: S1 Fig — The basemaps used in this study were sourced from the Department of Survey, Sri Lanka (https://survey.gov.lk/sdweb/home.php) for research purposes only. All rights remain with the Department of Survey, and any errors in interpretation or analysis are solely those of the authors. (TIF) [file pgph.0005192.s004.tif]
